# Open Versus Closed Reduction in Subtrochanteric Femur Fractures: The Critical Role of Acceptable Reduction in Healing Outcomes

**DOI:** 10.7759/cureus.99488

**Published:** 2025-12-17

**Authors:** Pedro Seabra, Daniel Gonçalves, Pedro Barra Simões, Margarida Gomes, Miguel Pimentel, José Oliveira, Henrique Sousa, André Sarmento

**Affiliations:** 1 Orthopaedics and Traumatology, ULSGE - Unidade Local de Saúde de Gaia/Espinho, Vila Nova de Gaia, PRT; 2 Orthopaedics and Traumatology, ULSAM - Unidade Local de Saúde do Alto Minho, Viana do Castelo, PRT; 3 Orthopaedics and Traumatology, Unidade Local de Saúde do Nordeste, Macedo de Cavaleiros, PRT

**Keywords:** fracture healing, intramedullary nailing, open and closed reduction, quality of reduction, subtrocantheric fractures

## Abstract

Introduction

Subtrochanteric femur fractures are challenging injuries due to high mechanical stress and compromised vascularization, resulting in higher complication rates compared with other proximal femur fractures. While intramedullary nailing is the treatment of choice, controversy persists regarding whether open or closed reduction provides superior outcomes. Some evidence suggests that open reduction may achieve better alignment, whereas others highlight risks of impaired biology, blood loss, and infection. This study aimed to compare open versus closed reduction in subtrochanteric fractures treated with intramedullary nailing and to assess the influence of reduction quality on bone healing outcomes.

Methods

We performed a retrospective cohort study including 190 patients with subtrochanteric fractures treated surgically between 2017 and 2024. After exclusions, 114 patients met the inclusion criteria (minimum six months of follow-up with complete clinical and radiographic data). Open reduction was defined as requiring an incision ≥3cm and the use of reduction instruments, with or without cerclage augmentation. Reduction quality was classified as acceptable or non-acceptable according to modified Baumgaertner criteria. Outcomes included delayed union (more than six months) and nonunion (more than nine months without progression). Statistical analyses included t tests, chi-square/Fisher’s exact tests, and Kaplan-Meier survival analysis, with significance set at p<0.05.

Results

Of the 114 patients, 39 (34.2%) underwent open reduction and 75 (65.8%) closed reduction. Acceptable reduction was achieved in 88 patients (77.2%), significantly more often in the open group (94.9% vs. 68.0%; p=0.001). The mean time to union was shorter in the open reduction group (18.2 ± 7.5 vs. 21.2 ± 7.9 weeks), but this difference did not reach significance (p=0.055). Delayed union occurred in 22 patients (19.3%), without significant group differences (12.8% open vs. 22.7% closed; p=0.21). Nonunion was observed only in the closed reduction group (5.3%; p=0.14). Reduction quality was a strong predictor of outcomes: the group with acceptable reduction had a faster union (18.1 ± 6.5 vs. 28.3 ± 7.6 weeks, p<0.001), lower delayed union (10.2% vs. 50.0%; p<0.001), and absence of nonunion (0% vs. 15.4%; p=0.002) in comparison with the non-acceptable reduction group.

Discussion

Although open reduction achieved acceptable alignment more frequently, clinical outcomes such as delayed union and nonunion did not significantly differ between open and closed approaches. Instead, reduction quality emerged as the most decisive factor, strongly associated with faster healing and fewer complications. These findings suggest that achieving an acceptable reduction outweighs the influence of the chosen surgical approach.

Conclusion

In subtrochanteric femur fractures treated with intramedullary nailing, reduction quality is the key determinant of healing outcomes. Acceptable reductions, whether achieved through open or closed techniques, significantly reduce delayed union and nonunion. Surgeons should prioritize achieving acceptable alignment.

## Introduction

Demographic changes with over-aging of societies are raising interest in the elderly population and the treatment of their pathologies [1]. Proximal femur fractures remain the most typical fragility fracture in this population and they are associated with high morbidity and mortality [1-3]. These fractures have a bimodal distribution and occur as a result of high-energy trauma in young and low-energy trauma in the elderly [4-6]. By definition, subtrochanteric fractures affect the region up to 5cm from the lesser trochanter and despite being less common they are associated with worse outcomes and higher complication rate [2,4,6-8]. That occurs due to great mechanical forces, caused by strong muscles acting as deforming forces and a precarious femur vascularization in this region leading to complications such as non-union, delayed union, mal-union and implant failure [2,4,5,7-9]. Although there is still debate on the definition of these terms, for most authors delayed union is defined as the absence of consolidation in the first six months and non-union when there is no consolidation after nine months and no signs of healing progression over the course of three consecutive months [2,8-10].

Despite all the evolution in surgical techniques and materials, the re-operation rate in these fractures still ranges around 10% [7-9] and the overall complication rate can be as high as 29% [5], with non-union reaching 20% [5,7]. There is a consensus that intramedullary nail is the best way to treat these fractures [2,4,6,7,9,11] and that at least we should have a 5cm fracture-screw distance as working length [5]. However there is still debate about whether open or closed reduction is better, both having advantages and disadvantages. Some authors advocate that with open reduction it is possible to obtain a more anatomical reposition improving stability and leading to lower non-union rates [2,4], but others reported higher non-union rates due to disturbances in biology and vascularization with consequences to fracture healing [12,13]. Besides that, open reduction is also associated with more blood loss, longer surgery time and soft tissue problems such as a higher rate of infection [2,4,7,8,14].

The main objective of this study was to evaluate whether open reduction had better outcomes when compared to closed reduction regarding delayed union and non-union and to establish the importance of achieving an acceptable reduction in treatment outcomes.

## Materials and methods

**Figure 1:**
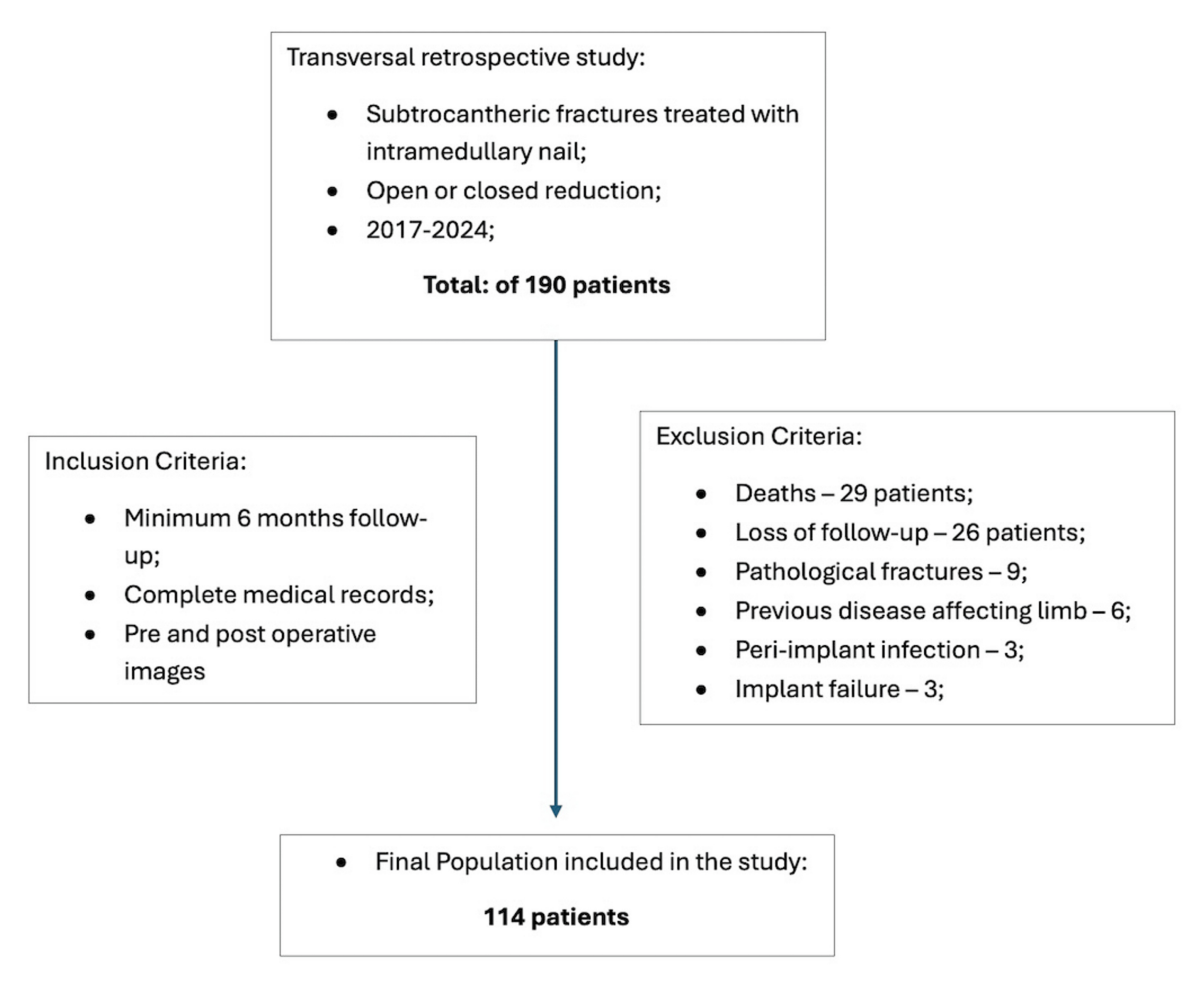
Cohort flowchart with inclusion and exclusion criteria - final population of 114 patients included in this study.

This transversal retrospective study included 190 patients with subtrochanteric fractures treated with open or closed reduction and intramedullary nail at our institution between 2017 and 2024. Inclusion criteria were a minimum of six months of follow-up and complete medical records, including pre- and postoperative images. Exclusion criteria were less than six months of follow-up (death or loss of follow-up); pathological fracture; previous disease affecting that limb; peri-implant infection; implant failure and peri-implant fracture. After applying exclusion criteria our final population was 114 patients (Figure 1).

Age, gender, previous functional status and type of approach (open vs closed reduction) were obtained from patient records. Open reduction was defined as the use of an incision of at least 3cm with use of reduction instruments, with or without cerclage augmentation. Percutaneous reduction was included in the closed reduction group. Acceptable reduction was defined according to modified Baumgaertner criteria in the post-operative AP and lateral X-rays and measured by two orthopedic surgeons as used in other similar studies [4,7-9]. Those surgeons were blinded to the surgical approach. Reduction was classified as non-acceptable when neither less than 10º angulation or less than 4mm deviation on both incidences was achieved. Consolidation was assumed when three out of four cortices had a bony bridge as suggested by Gomez-Barrena et al. [10]. Delayed union was considered when fractures healed after six months. Pseudarthrosis was defined as the absence of fracture healing in the first nine months associated with no signs of healing progression over the course of three consecutive months, which was in line with the US Federal Drug Administration's definition for non-union [10,15] and also in line with timings used in previous studies in order to be able to compare outcomes [2,8,9].

All analyses were performed using IBM SPSS Statistics, version 30 (IBM Corp., Armonk, NY, USA). Continuous variables were expressed as means and standard deviations and compared between groups using independent-samples t tests after assessment of homogeneity of variances with the Levene test. Categorical variables were summarized as frequencies and percentages, and associations with reduction type were assessed using the chi-square test (or Fisher’s exact test when expected counts were <5). For time-to-event outcomes (to evaluate time to fracture union distributions between groups), a Kaplan-Meier survival analysis with Log Rank (Mantel-Cox) test was applied. A p-value <0.05 was considered statistically significant.

This specific study protocol was approved by the relevant ethics committee and all patients gave full informed consent regarding their participation in this study.

## Results

Between 2017 and 2024, 190 patients suffered a subtrochanteric fracture that required surgical treatment at our institution. Thirty-three died (17% mortality during follow-up), 29 were lost to follow-up (15%), six had pathological fracture, two were excluded due to peri-implant infection, four due to implant failure, one due to peri-implant fracture and one for previous disease affecting the fractured limb. From those excluded, the two peri-implant infections and the peri-implant fracture had open reduction and all four implant failures had closed reduction, three of them with non-acceptable criteria.

A total of 114 patients were included: 39 (34.2%) underwent open reduction and 75 (65.8%) were treated with closed reduction. One hundred percent of surgeries were performed with long nails. The mean age in the open reduction group was 80.6 ± 11.7 years, compared with 78.6 ± 12.4 years in the closed reduction group. This difference was not statistically significant (t(112)=0.82, p=0.42; mean difference 2.0 years, 95% CI -2.8 to 6.7). Pre-injury ambulation was documented for 113 patients (99.1%). Of these, 82 (72.6%) were independent, 27 (23.9%) required external support, and four (3.5%) were bedridden. The distribution of pre-injury functional status did not differ significantly between open and closed reduction groups (χ²=3.26, p=0.20) (Table 1).

**Table 1:** Baseline and perioperative characteristics according to reduction method

	Overall (n=114)	Open reduction (n=39)	Closed reduction (n=75)	Test statistic	p value
Age, years (mean ± SD)	79.3 ± 12.1	80.6 ± 11.7	78.6 ± 12.4	t(179) = 0.94	0.42
Pre-injury ambulation, n (%):				χ² = 1.32	0.2
Independent	82 (72.6)	26 (66.7)	56 (75.7)		
Assisted	27 (23.9)	10 (25.6)	17 (23.0)		
Bedridden	4 (3.5)	3 (7.7)	1 (1.4)		

Open versus closed reduction

All patients were assessed for reduction quality. An acceptable reduction was achieved in 88 patients (77.2%), while 26 (22.8%) had a non-acceptable reduction. Acceptable reductions were significantly more frequent in the open reduction group (37/39; 94.9%) compared with the closed reduction group (51/75; 68.0%) (χ²=10.5, p=0.001) (Table 2).

**Table 2:** Comparison between open and closed reduction regarding outcomes WB - weight bearing

	Overall (n=114)	Open reduction (n=39)	Closed reduction (n=75)	Test statistic	p value
Reduction quality acceptable, n (%)	88 (77.2)	37 (94.9)	51 (68.0)	χ²=10.5	<0.001
Immediate postoperative WB, n (%):				χ²=5.59	0.06
Full weight-bearing	100 (87.7)	35 (89.7)	65 (86.7)		
Restricted	10 (8.8)	1 (2.6)	9 (12.0)		
Non-ambulatory prior	4 (3.5)	3 (7.7)	1 (1.3)		
Time to union, weeks (mean ± SD)	20.3 ± 7.9	18.2 ± 7.5	21.2 ± 7.9	t(108)=–1.94	0.055
Delayed union, n (%)	22 (20)	5 (12.8)	17 (22.7)	χ²=1.60	0.21
Nonunion, n (%)	4 (3.5)	0 (0.0)	4 (5.3)	χ²=2.16	0.14

Postoperative weight-bearing protocol was available for all 114 patients. Overall, 100 patients (87.7%) were allowed immediate full weight-bearing, 10 (8.8%) were restricted, and four (3.5%) had not been ambulatory prior to surgery. The difference between open and closed reduction groups approached but did not reach statistical significance (χ²=5.59, p=0.06) (Table 2).

The mean time to union was 20.3 ± 7.9 weeks overall. Patients treated with open reduction achieved union at a mean of 18.2 ± 7.5 weeks, compared with 21.2 ± 7.9 weeks in the closed reduction group. This difference was borderline non-significant (t(108)=-1.94, p=0.055; mean difference -3.0 weeks, 95% CI -6.1 to 0.06) (Table 2).

Delayed union was observed in 22 patients (20%), while 88 patients (80%) consolidated within the expected timeframe. The frequency did not differ significantly between groups (χ²=1.60, p=0.21): five patients (12.8%) after open reduction and 17 patients (22.7%) after closed reduction (Table 2).

Nonunion occurred in four patients (3.5%), all within the closed reduction group (5.3%). No cases of nonunion were observed after open reduction. This difference did not reach statistical significance (χ²=2.16, p=0.14) (Table 2).

Acceptable reduction and outcomes

Among patients with an acceptable reduction, 80 (90.9%) were allowed immediate full weight-bearing, compared with 20 (76.9%) of those with a non-acceptable reduction. The association between reduction quality and postoperative protocol was statistically significant (χ²=9.49, p=0.009), indicating that patients with better reductions were more frequently permitted immediate weight-bearing (Table 3).

**Table 3:** Association between reduction quality and outcomes WB - weight bearing

	Acceptable reduction (n=88)	Non-acceptable reduction (n=26)	Test statistic	p value
Immediate weight bearing, n (% full WB)	80 (90.9)	20 (76.9)	χ²=9.49	0.009
Delayed union, n (%)	9 (10.2)	13 (50.0)	χ²=20.4	<0.001
Time to union, weeks (mean ± SD)	18.1 ± 6.5	28.3 ± 7.6	t(108)=6.32	<0.001
Nonunion, n (%)	0 (0.0)	4 (15.4)	χ²=14.0	<0.001

Regarding bone healing outcomes, patients with an acceptable reduction consolidated significantly faster than those with a non-acceptable reduction. Mean time to union was markedly shorter with acceptable reductions (18.1 ± 6.5 vs. 28.3 ± 7.6 weeks, mean difference 10.2 weeks, 95% CI 7.0-13.4, p<0.001). Delayed union occurred significantly more often in patients with a non-acceptable reduction (13/26; 50.0%) compared with those with an acceptable reduction (9/88; 10.2%). This difference was highly significant (χ²=20.4, p<0.001), showing that poor reduction quality was strongly associated with delayed healing (Table 3).

Non-union occurred exclusively in patients with a non-acceptable reduction (4/26; 15.4%), while none of the patients with an acceptable reduction developed nonunion. This difference was statistically significant (χ²=14.0, p<0.001; Fisher’s exact p=0.002) (Table 3).

## Discussion

Subtrochanteric fractures are a special type of proximal femur fractures due to mechanical and vascularization issues that lead to higher rate of complications [2,4,5,7-9]. There is still considerable variability among studies regarding population age, high-energy vs. low-energy trauma, and the definition of open or closed reduction. Regarding age, there are studies where the mean age was 49.84 years old [4] and others with an older population [2,8,9,16], similar to our study. The definition of open and closed reduction is also not well established as many studies use terms like closed reduction, minimally invasive techniques and open reduction without defining them [17]. For example Hoskins et al. [7] were not able to analyse if in the control group (closed reduction) fracture was reduced by closed means or if reduction ocurred with open apporach but without cerclage augmentation. In our study open reduction was defined as the use of an incision of at least 3cm with use of reduction instruments, with or without cerclage augmentation, as we believe that all the disadvantages of open reduction are met even when cerclage wire is not used. These differences in definitions and study populations may result in comparisons across heterogeneous cohorts, making it more challenging to draw valid conclusions. Therefore, further research on this type of fracture remains pertinent, given its rising incidence and the need for more robust evidence.

In our study, regarding age and pre-injury status, there was no statistically significant difference between open and closed reduction groups, meaning that they are comparable when taking those variables into account. To define quality of reduction, especially for subtrochanteric fractures, the Baumgaertner et al. [18] criteria is a very useful classification. We used the modified criteria used in other studies [4,7-9] but we decided to divide only into two groups to emphasize the importance of obtaining at least an acceptable reduction. This means that when neither less than 10º angulation or less than 4mm deviation on both incidences was achieved reduction was classified as non-acceptable. On the other hand it is only necessary that angulation or deviation criteria be met to consider the reduction acceptable.

When comparing open to closed reduction we found a statistical difference in achieving an acceptable reduction (p<0.01) but no difference for the other four variables. That was in line with the systematic review from Hoskins et al. [7]. In their review, although there was only significant difference for the quality of reduction, there was a trend favouring open reduction for all other variables including non-union and time to union even with the open reduction group having a higher percentage of high-energy trauma. Many other studies such as Bonfiglio et al. [2] or Codesido et al. [8] also found significant difference regarding quality of reduction favouring open reduction, but as said before it is crucial to take into account the definition used for closed and open reduction in every study, as Teng Ma et al. [14] showed that percutaneous techniques can have same results as open reduction, regarding quality of reduction.

For post-operative protocol and time until union we found borderline non-statistical difference (p=0.06 for both variables) in favour of open reduction. Time until union was comparable with other studies for the open reduction group [4,8]. In a very similar population, Bonfiglio et al. [2] and Codesido et al. [8] found statistically difference in time to union with less time to union in the open reduction group, which is in line with our results. We believe that if we had a larger sample in the open reduction group that difference could be significant. 

Delayed union and non-union had no statistical difference between groups (p=0.21 and p=0.14 respectively) in our study. As mentioned before, for non-union a systematic review from 2021 also didn't find a statistical difference but there was a trend favouring open reduction [7], same result as Panteli et al. [19], opposing to Bonfiglio et al. [2] that had a significant difference between groups, with non-union occurring more in the closed reduction group. We believe that in our study it was not statistically different mainly due to sample size. Despite the non-statistical difference for delayed union and non-union, we should keep in mind that open reduction had significantly better quality of reduction as mentioned before, which in turn showed a significant difference compared to non-accecpable reduction both for delayed union and non-union.

Comparing the two groups for quality of reduction (acceptable vs non-acceptable reduction) we found a statistically significant difference for all four variables. That highlights the extreme importance of reaching a good reduction to achieve better outcome, as described in other studies [2,11,16,19]. Freigang et al. [20] even concluded that quality of reduction and valgization were the key factors to achieve union in their study.

Although the postoperative protocol depends on the surgeon, it seems reasonable that better reduction is adequate to resist deformation and compression, providing greater confidence for early weight-bearing [4,9]. The difference between the two groups was statistically significant (p=0.09) for post-op weight-bearing protocol. Time until union, delayed union, and nonunion were all statistically significant in favor of the group with acceptable reduction (p<0.01). Codesido et al. [9] and Polo et al. [5] divided their population into three groups regarding reduction (good, acceptable and poor). As in our study, they found that better reduction leads to less time to union and fewer complications such as delayed and non-union with statistical difference. Furthermore, Krapinger et al. [21] found that open reduction and cerclage wires are not risk factors for non-union, opposing to malreduction. In our study, although we have gathered acceptable and good reduction into the same group, it seems that it does not change the results. We can argue that when we achieve an acceptable reduction with closed methods there is no need for an open reduction, but we need more studies to prove that. These results are particularly important because it demonstrates that outcomes are improved when an acceptable reduction is achieved, regardless of whether it is obtained through open or closed reduction.

This study has several limitations: it is a retrospective study, which precludes control over radiographic assessments and their timing. In addition, the surgeries were performed by different surgeons, and the rehabilitation protocol was surgeon-dependent. There are also numerous other variables that may influence the bone healing process and were not accounted for, such as trauma energy, nail dynamization and presence of other medical comorbidities. Regarding time to union, it is difficult to know exactly the timing, as union can have occurred previously to the appointment, and even with good X-rays it can be quite difficult to assess, mainly in the open reduction group, as a more anatomic reduction can make it difficult to define the bony calus. We think that in future studies it would be important to clarify definitions of open and closed reduction, eventually comparing three groups: closed reduction, percutaneous reduction and open reduction.

## Conclusions

Open reduction was associated with a higher likelihood of achieving an acceptable reduction compared with closed reduction; however, no statistically significant differences were found between the two techniques in terms of delayed union or nonunion. Importantly, reduction quality itself was the strongest predictor of outcomes: patients with acceptable reductions had shorter time to union and significantly lower rates of delayed union and nonunion. These findings highlight that achieving an acceptable reduction is paramount for optimizing outcomes in subtrochanteric femur fractures, regardless of whether it is obtained through an open or closed approach. 
